# Flexible Holographic Fabrication of 3D Photonic Crystal Templates with Polarization Control through a 3D Printed Reflective Optical Element

**DOI:** 10.3390/mi7070128

**Published:** 2016-07-21

**Authors:** David Lowell, David George, Jeffrey Lutkenhaus, Chris Tian, Murthada Adewole, Usha Philipose, Hualiang Zhang, Yuankun Lin

**Affiliations:** 1 Department of Physics and Center for Advanced Research and Technology, University of North Texas, Denton, TX 76203, USA; DavidLowell@my.unt.edu (D.L.); davidgeorge2@my.unt.edu (D.G.); jeff.lutkenhaus@gmail.com (J.L.); chris.shang.tian@gmail.com (C.T.); murthadaadewole@my.unt.edu (M.A.); usha.philipose@unt.edu (U.P.); 2 ECE Department, University of Massachusetts Lowell, Lowell, MA 01854, USA; hualiang_zhang@uml.edu; 3 Department of Electrical Engineering, University of North Texas, Denton, TX 76203, USA

**Keywords:** micro/nano fabrication, 3D laser fabrication, holographic lithography, single optical element, photonic crystals

## Abstract

In this paper, we have systematically studied the holographic fabrication of three-dimensional (3D) structures using a single 3D printed reflective optical element (ROE), taking advantage of the ease of design and 3D printing of the ROE. The reflective surface was setup at non-Brewster angles to reflect both s- and p-polarized beams for the interference. The wide selection of reflective surface materials and interference angles allow control of the ratio of s- and p-polarizations, and intensity ratio of side-beam to central beam for interference lithography. Photonic bandgap simulations have also indicated that both s and p-polarized waves are sometimes needed in the reflected side beams for maximum photonic bandgap size and certain filling fractions of dielectric inside the photonic crystals. The flexibility of single ROE and single exposure based holographic fabrication of 3D structures was demonstrated with reflective surfaces of ROEs at non-Brewster angles, highlighting the capability of the ROE technique of producing umbrella configurations of side beams with arbitrary angles and polarizations and paving the way for the rapid throughput of various photonic crystal templates.

## 1. Introduction

Photonic crystals (PhCs) are photonic nano/micro-structures in which the refractive index is periodically modulated. For three-dimensional (3D) PhCs, stop bands will appear in certain symmetrical directions. The stop bands can overlap to form a full photonic band gap if the refractive index contrast is large enough in 3D PhCs with certain crystal symmetry. Three-dimensional PhCs have amazing optical applications in ultra-high Q optical resonators for lasers [1,2], optical cloaking devices [3], and photon conversion efficiency enhancement in solar cells [4]. In addition, 3D photonic structures can be fabricated by layer-by-layer micro-fabrication [1], self-assembly of colloidal spheres [5,6], and direct laser writing (DLW) [3,7,8,9,10]. DLW is a very attractive process as it has demonstrated the capability of fabricating periodic, quasi-periodic, and graded refractive index structures [3,7,8,9,10]. However, DLW is a serial process, thus taking several hours for small volume structures.

Interference based holographic lithography has been used for the parallel and simultaneous fabrication of large area (volume) photonic structures by one exposure process [11,12]. Theoretically, the holographic lithography approach can produce 3D photonic structures with all symmetries defined by the Bravais lattices [13]. The multi-beam interference patterns (and therefore formed holographic structures) can be controlled by the number of interfering beams, beam polarizations, intensity ratio among beams, phase of the beams, angles of interference and iso-intensity threshold [14]. Conventional holographic lithography setups need to use bulk optical elements to control these parameters [11,12]. These bulk optical elements can occupy half of a 4 × 8 ft^2^ optical table, making mechanical stability a problem. Recently, the use of single diffractive [14,15,16,17,18,19,20,21,22,23] and reflective [24,25,26,27,28,29,30] optical elements has significantly simplified the optical setup for holographic lithography. By this method, 3D PhCs have been fabricated through a single beam, single optical element, and single exposure process [16,17,18,19,20,21,22,23,24,25,26,27,28,29,30]. In highly symmetric 3D PhCs, the photonic band structures are more isotropic (the Brillouin surface is more spherical), thus it is much easier to achieve a full photonic band gap than in those with lower crystal symmetry [14,28]. Using a single beam and single diffractive element, all diffracted beams come from the same half space, thus the lattice periodicity in the *z*-direction is larger than the periodicity in the *xy*-plane. A multi-level phase mask has been designed for the holographic fabrication of diamond-like PhCs [14]. However the fabrication of 3D PhCs with similar lattice constants in all directions has not been realized yet using multi-level phase masks [20,21,22,23]. In addition, the relative rotation of two gratings away from 90 degrees can be used to adjust the periodicity difference in different directions and increase the photonic band gap [17]. On the other hand, reflective optical elements (ROEs) can be used to easily achieve the desired interference angles that produce 3D PhCs with diamond-like symmetry (woodpile structures) [24]. By using a single 3D printed ROE without any other bulk optics and a single circularly polarized beam, we have fabricated woodpile PhCs through the interference of reflected, s-polarized side beams together with a circularly polarized central beam [30].

Although the holographic fabrication of 3D structures has been reported when the reflective surfaces of ROEs were setup at the Brewster angle [30], we need to further understand the following factors when the reflective surfaces are setup at non-Brewster angles (thus p-polarizations are introduced into the interference): (a) how the photonic bandgap is affected if both s- and p-polarized beams are used for the interference; (b) what the filling fraction of photonic crystals is when the maximum bandgap size occurs; (c) whether the interference pattern is still bi-continuous so that the un-polymerized photoresist can still be developed out of the holographic structures; and (d) whether the ROE technique is flexible for the interference lithography with arbitrary angle and polarization. On the other hand, it is more difficult to holographically fabricate 3D structures with a small period than those with a big period.

In this paper, we systematically study the holographic fabrication of 3D PhCs using 3D printed ROEs, taking advantage of the ease of design and fabrication of the ROE. We study the interference lithography of 3D PhCs with the reflective surfaces setup at non-Brewster angles or deliberately placed at different angles. We study the effects on the photonic bandgap and the change of filling fraction in the structure if both s- and p-polarized side beams are used for the multiple-beam interference. We demonstrate that the interference pattern is still bicontinuous even if both s- and p-polarized side beams are used for the interference.

## 2. Experiments

**Figure 1:**
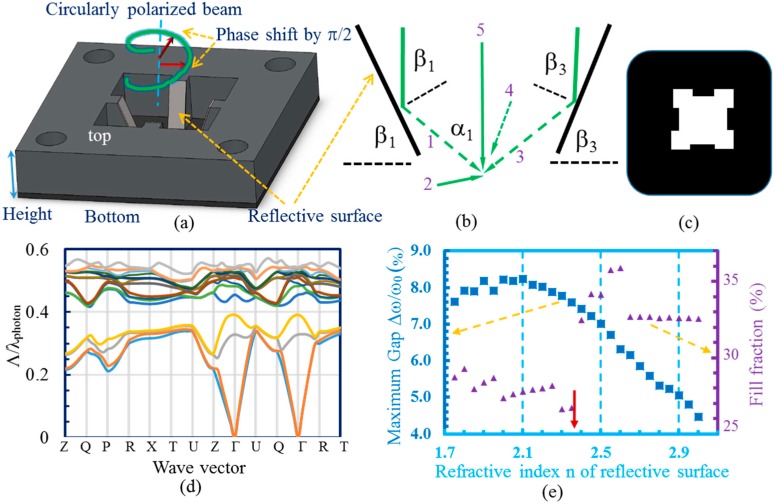
(**a**) Model of the lab-designed ROE in the 4 + 1 configuration, designed using CAD software. The reflective surfaces are mounted on the support structures to reflect a single circularly polarized laser beam; (**b**) schematic of wave vector configuration for four side beams and one central beam in the 4 + 1 configuration; (**c**) schematic of an aperture for beam selection; (**d**) photonic band structure for one of holographic photonic crystals; and (**e**) plot of maximum bandgap size and filling fraction of dielectric that produces maximum bandgap for the PhCs that can be fabricated using a reflective surface with refractive index n and angle of incidence 67°.

Three-dimensional designs of the ROE were generated using SolidWorks software (Student Edition, Dassault Systèmes, Vélizy-Villacoublay, France), and the ROEs were printed in acrylonitrile–butadiene–styrene (ABS) plastic using an UP! 3D printer (Tiertime Corporation, Beijing, China). Figure 1a shows a schematic of one of the ROEs with four reflecting surfaces. Polished silicon was cut into pieces and adhered to the plastic support to reflect the incident circularly polarized laser beam. As shown in the 4 + 1 umbrella setup in Figure 1b, four reflected beams overlap with the central circularly polarized beam to produce the interference. The circular polarization has an intrinsic phase shift of π/2 between orthogonal directions for the generation of woodpile structures using four reflective surfaces in the ROE. In addition, the intensity ratio of side beams is determined by the incident angle. The incident angle to the reflective surface is equal to the angle of the designed support structure (thus the reflective surface) relative to the horizontal plane. From the interference geometry in Figure 1b, the interference angle, α, between a side beam and the central beam is α = 180 − 2β. When the interference angle increases, the height of the ROE frame as indicated in Figure 1a needs to be reduced. The reflective surfaces were put closely together and the areal size of each reflective surface was reduced in order to illuminate all reflective surfaces under the single laser beam. Figure 1c shows an aperture for beam area selection for multi-beam interference. Without the aperture, only one exposure spot can be made on the 1 inch^2^ substrate, but with the aperture, four exposure spots are possible. Instead of putting it on top of the ROE [30], it was put at the bottom of the ROE (between the ROE and photoresist) in order to reduce the effects of diffraction.

Such an ROE has the following flexibility: (a) the reflective surface can be setup in a wide range of angles β. If β = 76.5, the reflective surface is setup at Brewster’s angle of silicon at 514.5 nm. If α = 70.5 degrees (corresponding to β = 54.75 degrees), an interference pattern with face-centered-cubic symmetry is generated by the 4 + 1 configuration [14,15,16]. Therefore, the reflective surface can be setup in the range of 54.75 and 76.5 degrees if silicon is used; (b) the reflective surface can be changed to have a refractive index n and a Brewster’s angle at a desired angle of incidence, or moved away from Brewster’s angle. When not at Brewster’s angle, the reflected beams have both s- and p-polarized components. Different ratios of s- and p-polarized components will result in different patterns (or motifs) in the unit cell of the interference pattern and can be used to optimize the band gap size [14]; (c) the number of reflective surfaces can easily be changed by the designer; and (d) each reflective surface can be setup at different angles.

An Ar ion laser 514.5 nm line (Innova Sabre, Coherent Inc., Santa Clara, CA, USA) was used for the holographic fabrication of PhCs with an expanded beam diameter of about 5 cm. The holographic structures were recorded in a modified negative photoresist SU-8 (MicroChem Corp., Westborough, MA, USA) [30]. Photosensitizer H-NU 470 (Spectra Group LTD. Inc., Millbury, OH, USA) was added to SU-8 to make an SU-8 mixture that is sensitive to the laser beam at 514.5 nm. The modified SU-8 mixture was spin coated on clean glass slides at 700 rpm for 30 s and baked on a hotplate at 65 °C for 90 min to remove the solvent. A typical laser power of 3.0 W was used and exposure times for good samples were in a range from 3 to 5 s. Post-exposure baking time was 40 min at 65 °C. Samples were developed in propylene glycol monomethyl ether acetate (PGMEA) for 12 h and rinsed in isopropyl alcohol for 30 s after development. For the purpose of quick testing of the ROE setup, dipentaerythritol hexa/penta-acrylate (DPHPA) mixture [31] was used for an initial exposure.

## 3. Simulation Results

The photonic bandgap size is mainly determined by the interference angle [14,15,16]; in order to understand the effect of just including the p-wave in the interference, we run simulations keeping the same interference angle but changing the refractive index n of the reflective surface, although it is hard to smoothly vary n in practice. To be general, we calculated the photonic band gap using MIT’s Photonic Bands (MPB) package (ver. 1.4.2, Cambridge, MA, USA) [32] for the interference pattern formed by a 4 + 1 configuration with varying reflecting surfaces and the same angle of β = 67 degrees (the interfering angle relative to central beam is 46 degrees) for each reflective surface. Usually, the holographic structure is used as the photonic crystal template and materials with high dielectric constant will replace the polymer through an inversion or double-inversion [5]. Using MATLAB (ver. R2012b, Natick, MA, USA), Linux (Ubuntu 10.04, London, UK), Python (ver. 2.6.5, Wilmington, DE, USA), Octave programs (ver. 3.2.3, Boston, MA, USA), and MPB simulations, the interference data was generated in a discretized grid, and then converted to a 3D dielectric function by replacing the values of intensity in the discretized grid with values for the dielectric constant at that point. At points where the intensity was higher than a threshold intensity value (simulating the development process), then the intensity value was replaced with 11.9, to represent silicon. Every point that had intensity lower than the threshold was replaced with a value of 1, representing air. The high symmetry *k*-points as labeled on the *x*-axis in Figure 1d on the Brillouin surface for the holographic structures were calculated [16,17]. For β = 67 degrees, the photonic band structure (one example is shown in Figure 1d) was calculated and the photonic band gap can be obtained from the photonic band structure in Figure 1d. The results are summarized in Figure 1e. The *x*-axis is the refractive index of the material used for the four reflective surfaces. The red solid arrow indicates the location of *n* = 2.36, the material with Brewster’s angle of 67 degrees. For each refractive index n used for the reflective surfaces, various iso-intensity surfaces of the interference pattern (thus various filling fractions) were input into MPB to search for a maximum photonic band gap size. The maximum band gap size in solid squares (and the corresponding filling fraction in solid triangles) is plotted on the *y*-axis of Figure 1e. As seen from the figure, the maximum bandgap occurs when *n* = 2.0, which is not the index of refraction of the material with Brewster’s angle of 67 degrees. The maximum bandgap occurs when both s- and p-polarized beams are included in the side beams of the multiple-beam interference. The filling fraction of maximum photonic bandgap structures is lower to the left of the red solid arrow than simulated structures to the right of the red arrow. Applications that require small filling fractions (thus large pore size) and large bandgaps, such as photonic crystal-based dye-sensitized solar cells [4], would use a reflecting surface with index of refraction 2.0.

## 4. Experimental Results

### 4.1. Fabrication of 3D Structures through Multi-Beam 4 + 1 Interference

Our previous study has fabricated woodpile PhCs with the reflective surfaces placed at Brewster’s angle in the 4 + 1 configuration and at the same angle of incidence for all reflective surfaces [30]. The reflected s-polarized beams (1 and 3) do not interfere with beams (2 and 4) as labelled in Figure 1b. In this paper, the reflective surfaces are placed away from Brewster’s angle. The interference of four s-polarized side beams and one central circularly polarized beam has a woodpile structure [30], while the interference of p-polarized side beams with a central circularly polarized beam produces ellipsoidal structures [15] (also shown in the discussion section). Furthermore, we deliberately placed one reflective surface at an angle different from the others. The interfering angles relative to the central beam were (α_1_, α_2_, α_3_, α_4_) = (46, 46, 43, 46) degrees. Polished silicon chips were used again as the reflective surfaces thus the intensity ratio of s-wave to p-wave was approximately 100:9 in each reflected beam.

When multiple beams are overlapped, the intensity distribution of their interference pattern can be determined by the following equation:

(1) $$I=\langle \sum_{i=1}^{n}E_{i}^{2}\rangle +\sum_{i<j}^{n}E_{i}E_{j}e_{i}\cdot e_{j}\cos\left[\left(k_{i}-k_{j}\right)\;r+\Delta \delta_{i\;j}\right],$$

where *E*, *e*, and δ are the electric field, polarization direction, and initial phase, respectively, for wave vector *k*, and *n* = 5 for 4 + 1 interference. The wave vectors in Equation (1) for the central and side beams can be described by:

(2) $$k_{0}=K(0,\;0,\;1),\;k_{1}=K(\sin46,0,\;\cos46),\;k_{2}=K(0,-\sin46,\;\cos46),\;k_{3}=K(-\sin43,0,\;\cos43),\;k_{4}=K(0,\sin46,\;\cos46),$$

where *K* = 2π/λ.

**Figure 2:**
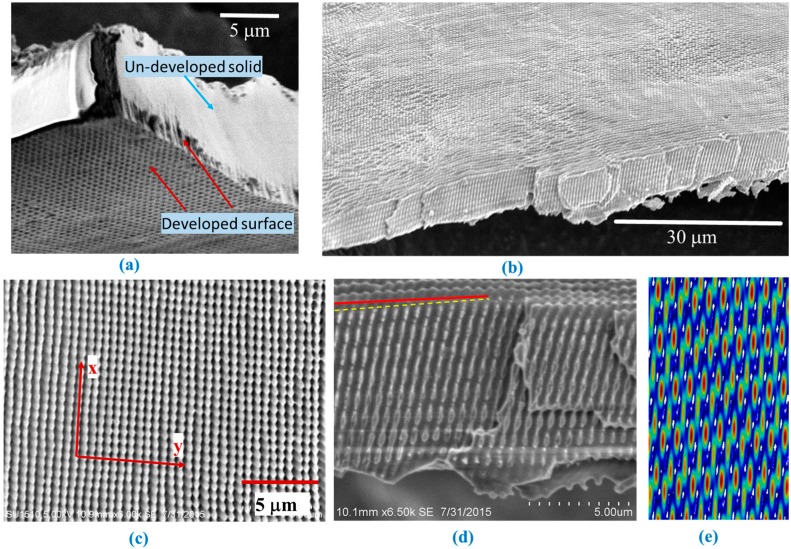
(**a**) Cross-section view SEM of over-exposed sample formed with 4 + 1 configuration ROE; (**b**,**c**) Top-view SEM of holographically fabricated, well-developed 3D structures with 4 + 1 configuration ROE with large areas showing no diffraction pattern (b) and enlarged view (c); Fabricated (**d**) and (**e**) simulated cross-sectional view of 3D structure formed with 4 + 1 configuration. The structure is not uniform as was expected due to a designed shift in the angle of incidence of a side beam. The dark-red solid line is in parallel with the sample surface and the yellow dashed line indicates the orientation of the holographic structures.

During the interference lithography, the effects of diffraction from the beam aperture were studied in the over-exposed sample as shown in Figure 2a. A perfect 2D periodic structure, labeled as developed surface in Figure 2a, can be obtained without ring structures if diffraction was reduced by painting the reflective surface edge with black marker. Figure 2b shows a large-area SEM of a well-developed sample after removing the diffraction. Some irregular spots are due to the quality of polishing of the raw silicon wafers. Structures with many more layers in the *z*-direction and smaller periodicity than those in a previous study [30] were obtained. Figure 2c,d show the enlarged top and cross-sectional views of fabricated structures in SU-8, respectively. From the top-view, the ellipsoid-like surface is evident due to the contribution from p-wave [15]. The periodicity on the *y*-axis as shown in Figure 2c is 668 nm as measured by the AFM. The theoretical periodicity is (514.5/sin46) = 715 nm. The average *x*-direction periodicity is measured to be 690 nm by AFM. Theoretically, we assume it is the average of (514.5/sin46) = 715 nm and (514.5/sin43) = 754 nm, which is 730 nm. The smaller measured value might be due to sample shrinkage. Figure 2d,e show an scanning electron microscope (SEM) in cross-section of the *xz*-plane of the fabricated structures and the simulated interference pattern, respectively. The solid red line is parallel with the PhC surface and the yellow dashed line is rotated by 1.5 degrees, showing that the fabricated structure on the *xz*-plane is rotated by 1.5 degrees. The structure in Figure 2d is not uniform as was simulated in the insert due to the designed shift in the angle of incidence of a side beam. Disorders have been deliberately incorporated into multi-lattice PhCs for solar cell applications [33,34]. The average periodicity in the *z*-direction is measured to be 1967 nm, very close to the theoretical calculation (514.5/(1 − cos43)) = 1915 nm but larger than the (514.5/(1 − cos46)) = 1686 nm. The above study indicates the fabrication capability of 3D structures even when the p-polarized wave is included in the interference. Furthermore, it is possible to fabricate superlattice PhCs where four side beams have interfering angles (α_1_, α_2_, α_1_, α_2_) relative to the central beam (α_1_ ≠ α_2_).

### 4.2. Fabrication of Quasi-Crystals through Multi-Beam 5 + 1 Interference

For quasi-crystal fabrication, an ROE was 3D printed with 67 degree support structures in a 5 + 1 interference configuration. Silicon chips were used as the reflective surfaces to test the capability of holographic fabrication quasi-crystals when both s- and p-polarized beams are involved in the interference lithography. The side beams have wavevectors:

(3) $$k_{q}=K(\sin46\;\cos\frac{2(q-1)\pi }{5},\;\sin46\;\sin\frac{2(q-1)\pi }{5},\;\cos46),\;q=\text{1, 2,}...\text{,5},$$

where *K* = 2π/λ. The wave vector differences Δ*k* can be considered as reciprocal lattice vectors of holographically formed structures. The first-order wave-vector difference Δ*k_first_* between the neighboring side beams can be described as

(4) $$\Delta k_{first}=k_{q}-k_{q-1}=2K\;\sin46\;\cos(\frac{3\pi }{10})(\cos(\frac{2q}{5}-\frac{1}{10})\pi ,\;\sin(\frac{2q}{5}-\frac{1}{10})\pi ,\;0).$$

**Figure 3:**
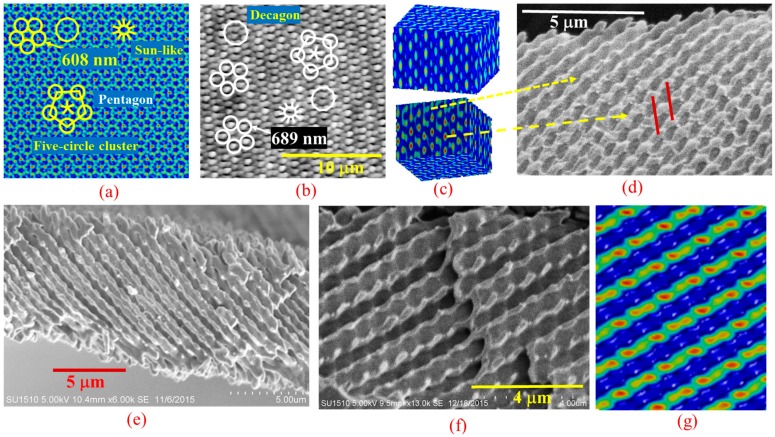
(**a**) Simulated top view of the 5 + 1 interference pattern and (**b**) atomic force micrograph (AFM) of fabricated quasi-crystals in DPHPA using a 5 + 1 configuration ROE. Five-circle clusters, pentagons, sun-like shapes of ten lines and decagons are drawn for eye guidance; (**c**) simulated interference patterns showing the front and back sides of a cubic volume; (**d**–**f**) cross-section SEM images of fabricated 3D quasicrystals in SU-8 cut in different orientation revealing various structural information; and (**g**) a side view of simulated 3D interference pattern due to 5 + 1 beams.

The 5 + 1 interference yields structures that are quasicrystalline in the *xy*-plane and periodic in the *z*-direction. The structure lacks translational symmetry in the *xy*-plane but has rotational symmetry. Calculation of Δ*k_first_* yields a radius of 2*K*sin46cos(3π/10) of a circle in k space, and in real space *R* = 2π/|Δ*k_first_*| = λ/(2sin46cos(3π/10)) is the radius of the circular holographic structures. Figure 3a,b show the simulated interference pattern and an AFM image of the fabricated quasicrystal in DPHPA, respectively. In reciprocal space, the five Δ*k_first_* between the five neighboring side beams are all equal. In the interference pattern, five circles form a cluster with a radius of *R* = 514.5/(2sin46cos(3π/10)) = 608 nm as indicated by the arrow in Figure 3a. *R* = 689 nm was measured by AFM in Figure 3b. Five-fold symmetry can be seen in both Figure 3a,b from the five-circle clusters, pentagons, decagons (10-gon), and sun-like shapes of ten lines. From the theory, the structure in the *z*-direction is periodic and the calculated periodicity is λ/(1 − cos(46)) = 1685 nm. Figure 3c shows the front and back sides of a cubic volume of the simulated interference pattern. The top figure revealed a cross-section where we can see the periodicity in the *z*-direction, while the bottom figure looks different from the top one because the interference pattern lacks translational symmetry in quasi-crystals. Figure 3d–f show cross-section SEM images of fabricated quasicrystals in SU-8 revealing various structural information. The bottom of Figure 3c is a simulated cross-section of the SEM in Figure 3d where the periodicity in the *z*-direction is indicated by the red lines. The periodicity was measured to be 1723 nm. The cross-section in Figure 3d was not uniform, as was simulated in the bottom of Figure 3c where dashed yellow lines point to their corresponding parts with high intensity and low-intensity regions. When cut along another direction, the cross-section of fabricated structure in Figure 3e shows channel-like structures. Multi-layer, channel-like structures were revealed in Figure 3f, which can be closely simulated in Figure 3g. All these results show that the holographic structures can still be developed out (i.e., they are bicontinuous) when including p-polarized waves in the interference.

### 4.3. Holographic Fabrication Using ROE for Multiple Beam 6 + 1 Interference

Ideally, we can get more isotropic PhCs with the 6 + 1 configuration than the 4 + 1 or 5 + 1 configuration because of the isotropic motif in the *xy*-plane of the 6 + 1 interference. We have not worked out the photonic band gap calculation yet for the holographic structures; however, we focus on the fabrication in this paper. Silicon chips were again used as the reflective surface and supported at the same angle of 67 degrees in an ROE with 6 + 1 configuration to test the capability of holographic fabrication of 3D hexagonal PhCs when both s- and p-polarized beams are involved in the interference lithography. The side beams have wavevectors:

(5) $$k_{q}=K\;(\sin46\;\cos\frac{2(q-1)\pi }{6},\;\sin46\;\sin\frac{2(q-1)\pi }{6},\;\cos46),\;q=\text{1, 2,}...\text{,6}.$$

The first-order wave-vector difference Δ*k_first_* between the neighboring side beams can be described as

(6) $$\Delta k_{first}=k_{q}-k_{q-1}=2K\;\sin46\;\cos(\frac{\pi }{3})(\cos\frac{n}{3}\pi ,\;\sin\frac{n}{3}\pi ,\;0).$$

**Figure 4:**
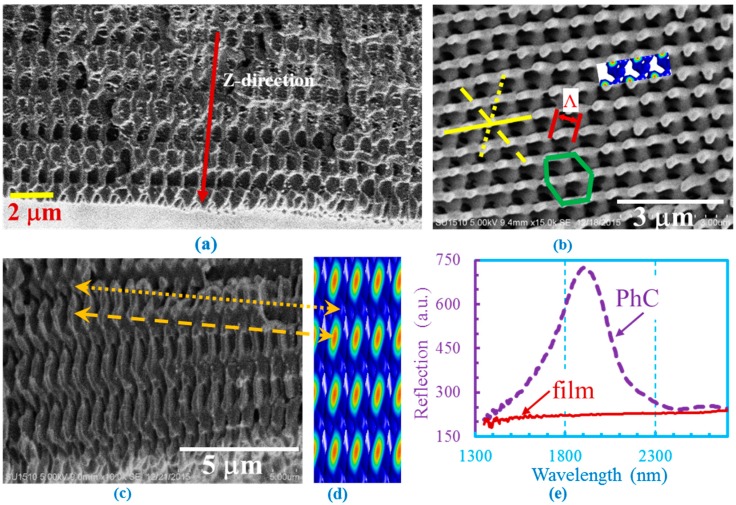
(**a**) SEM of cross-section of not fully developed 3D PhCs in SU-8 but cut in an orientation where layer-by-layer pattern can be seen; (**b**) SEM of top-view of fabricated structures with six-fold symmetry in SU-8. Yellow lines indicate the orientation of structures in different layers. The hexagonal structure and the lattice constant Λ are indicated by the green hexagon and red arrow, respectively, for eye guidance; (**c**) SEM of the cross section of fabricated 3D PhCs in SU-8 and (**d**) the simulated interference pattern. Yellow lines indicate analogous fabricated and simulated structures; (**e**) measured FTIR reflection spectra from SU-8 PhCs and overexposed SU-8 film without any structures.

The structure is periodic in the *z*-direction. Due to the six-fold rotational symmetry, it is not easy to cut a cross-section with nice layer-by-layer structures. In one of our samples as shown in Figure 4a, the structure was not well-developed but revealed layer-by-layer structures. The top-view of the fabricated sample shows hexagonal structures as seen in the SEM image of well-developed structures in SU-8 in Figure 4b. The hexagonal structure is indicated by the green hexagon. The lattice constant Λ as labelled in the Figure 4b was calculated by Λ = 2π/|Δ*k_first_*| = λ/(2sin46cos(π/3)) = 715 nm. From the SEM, Λ = 632 nm was measured.

Three yellow lines have been drawn for eye guidance in Figure 4b. The yellow dotted and dashed lines are rotated by 60 and 120 degrees from the yellow solid line, respectively, indicating six-fold symmetry of the structure. Figure 4c shows the SEM cross-sectional view of the same fabricated structures as in Figure 4b and their simulated patterns are shown in Figure 4d. Although the cross-section was not perfectly cut, analogous parts between the fabricated and simulated structures can be found in the figure as indicated by the yellow dotted and dashed lines. The periodicity in the *z*-direction is measured to be 1741 nm, compared with the theoretical periodicity of *c* = λ/(1 – cos(46)) = 1685 nm.

Figure 4e shows the reflection spectra from holographic structures in SU-8 PhCs from the (001) surface and an over-exposure SU-8 film (without 3D structures) measured by Fourier transform infrared (FTIR) spectroscopy. The reflection from the SU-8 film is a flat line; however, a reflection peak centered at 1929 nm was observed from the SU-8 PhCs. The reflection location can be estimated by:

(7) $$\lambda =c\sqrt{n_{SU-8}^{2}f_{SU-8}+n_{air}^{2}(1-f_{SU-8})}=1685\sqrt{1.6^{2}\times 0.2+1^{2}\times (1-0.2)}=1930\;nm,$$

where *c* is the periodicity in the *z*-direction, *n* the refractive index of SU-8 (*n* = 1.6 is estimated), and *f* is the filling fraction of dielectric SU-8 in PhCs. The filling fraction *f* of 20% in Equation (7) was used in order to obtain a wavelength of 1930 nm. The inset in Figure 4b is a simulated interference pattern with a filling fraction of 23.8%, fitting closely with the fabricated structure.

## 5. Discussion

The reflection peak in Figure 4e is quite broad. This can be attributed to the gradient filling fraction change along the *z*-direction as indicated in Figure 4a. Because we added photosensitizer H-NU470 (Spectra Group LTD. Inc., Millbury, OH, USA) (which has its absorption peak at 470 nm) into SU-8 mixture, the to-be-exposed sample absorbed more laser light at the surface than at the bottom. The bottom part of the sample has a lower filling fraction than that at the top part as shown in Figure 4a as an example. Based on Equation (7), the Bragg reflection will broaden because of the gradient filling fraction. Further simulation of reflection spectra from the holographic structures using MIT Electromagnetic Equation Propagation program (MEEP, ver. 1.3, Cambridge, MA, USA) can help understand the peak broadening. On the other hand, the gradient filling fraction in photonic crystals could be used to increase the photonic bandgap size or to realize transformation optics devices [35].

**Figure 5:**
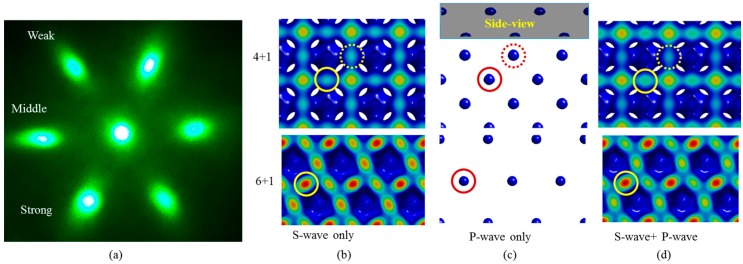
(**a**) Diffraction pattern from fabricated 3D photonic crystal template in SU-8 using 532 nm laser; (**b**) top-view of simulated interference among pure s-wave side beams plus the central circularly polarized beam; (**c**) among pure p-wave side beams plus the central circularly polarized beam; (**d**) among side beams with both s- and p-waves plus the central circularly polarized beam. Interference patterns generated with 4 + 1 and 6 + 1 configurations are shown in top and bottom row in (b,c,d), respectively. Circles indicate the corresponding locations of high intensity spots. Dotted circles indicate the spots located at the bottom as shown in the side view in the insert in (c).

The gradient filling fraction along the *z*-direction in fabricated photonic crystals with 6 + 1 configuration is also revealed in the diffraction pattern of the crystal by 532 nm laser. The pattern is shown in Figure 5a. The pattern has a pair of strong diffraction spots in one direction and diffraction spots look weaker and weaker in a direction rotated away by 60 and 120 degrees. The dotted and dashed yellow lines in Figure 4b indicate the structures rotated by 60 and 120 degrees, respectively, from the solid yellow line. After each rotation by 60 degrees, the structure moves deeper in the *z*-direction. The different diffraction efficiencies in different directions with 0, 60 and 120 degrees in Figure 5a correspond to the gradient filling fraction changes in the *z*-direction in the fabricated structures in Figure 4b,c. The diffraction pattern in Figure 5a is sharper than the one in reference 30 due to the presence of more layers of periodic structures in the *z*-direction in this paper. We could not tell from the diffraction pattern whether the p-wave will make any difference in the interference.

Figure 5b–d show simulations of interference patterns generated among the pure s-wave side beams + central circularly polarized beam, the pure p-wave side beams + central circularly polarized beam, and the combination of s-wave and p-wave side beams used in this paper + central circularly polarized beam, respectively. The top row in Figure 5b–d is the interference patterns generated by 4 + 1 configuration, and the bottom row are those generated by 6 + 1 configuration. The patterns were obtained using the same intensity threshold for each configuration. At 67 degrees and with silicon as the reflective surface, the intensity ratio of the s-wave to the p-wave is close to 11:1 when the circularly polarized beam is reflected. Thus, the interference patterns in Figure 5c is much weaker than that in Figure 5b. We see from these figures that the high intensity spots in Figure 5c are located at the high intensity spots in Figure 5b. Thus, the formed 3D holographic structures are still bi-continuous when both s- and p-waves are included for the interference lithography.

## 6. Conclusions

In summary, holographic fabrication of 3D photonic structures has been realized through a single circularly polarized beam and single ROE with reflective surfaces supported at non-Brewster angles. Photonic bandgap and filling fraction of dielectrics in 3D structures have been calculated for the holographic structures generated with a selection of refractive indices for the reflective surface, indicating the necessity of non-Brewster configuration in ROE for specific applications. Holographic fabrication capability and flexibility has been demonstrated for future arbitrary angle and polarization configuration by a wide selection of reflective surface materials and interference angles. Both experimental and simulation results have shown that the holographic structures are still bi-continuous when including p-polarized waves in the interference.
